# Improving Pasteurization to Preserve the Biological Components of Donated Human Milk

**DOI:** 10.3389/fped.2018.00288

**Published:** 2018-10-09

**Authors:** Antoni Gayà, Javier Calvo

**Affiliations:** ^1^Banc de Llet Materna, Fundació Banc de Sang i Teixits de les Illes Balears, Palma de Mallorca, Spain; ^2^Terapia Celular e Ingeniería Tisular, Institut d'Investigació Sanitària Illes Balears, Palma de Mallorca, Spain

**Keywords:** donated human milk, pasteurization, human milk bank, biological components of milk, temperature

## Abstract

Donor human milk (DHM) in human milk banks (HMB) is routinely subjected to heat treatment to ensure microbiological security, most guidelines recommending a temperature of 62. 5°C for 30 min. However, this procedure negatively impacts on milk quality, due to the destruction of biological components. Different studies have called for a more respectful treatment of DHM to preserve its properties, and have explored the use of alternative technologies. There is also clear evidence that bacterial and viral contamination in human milk can be effectively destroyed by temperatures lower than that currently recommended (62.5°C). Thus, a simple option would be to optimize the conventional pasteurization technique so the treated milk is free of infectious elements yet retains a maximum amount of biological components. An advantage of this approach is that it would be unnecessary to replace the pasteurization equipment currently available in most HMB. On the basis of a literature review, we here analyze and discuss evidence that pasteurization of human milk at a temperature below 62.5°C results in an improved preservation of its properties without compromising safety regarding the transmission of infectious agents.

## Introduction

There is general agreement that breastfeeding is the optimal nutritional source for infants (1, 2). Human milk is a synergistic package of essential nutrients and bioactive components that not only covers the nutritional needs of the neonate but also enhances host defenses against infection, actively modulating the immune response. Its consumption facilitates the maturation of various organs and neurological development, modifies the intestinal bacterial flora, and improves the digestion and absorption of nutrients. Beneficial bioactive and immunomodulatory constituents of breast milk include gastrointestinal hormones, immunoglobulins, lactoferrin, lysozyme, oligosaccharides, nucleotides, growth factors, enzymes, antioxidants, and cellular components (3, 4).

When the mother's own milk is not available, the WHO as well as most scientific associations consider DHM, obtained and processed in HMB, as the best alternative, especially in preterm infants (2). As a safety mechanism, HMB usually pasteurize the donated milk to eliminate infectious elements. The technique is named after Pasteur, who first described it in the XIX century as a way of preventing the souring of wine and beer and extending their shelf life. Pasteurization is defined as a process of heating a food, usually a liquid, at a specific temperature for a predefined length of time and then immediately cooling it. The crucial point of the procedure is the selected temperature, which should be high enough to destroy microbial contamination without affecting the properties of the food.

## Heat treatment of DHM

The use of pasteurization in HMB is based on the experience of the food industry with the treatment of cow's milk (5). Initially, pasteurization of cow's milk was carried out at 61.1°C for 30 min or 71.1°C for 15 s to allow an ample safety margin for the destruction of *Mycobacterium tuberculosis* (6). However, in 1957 these conditions were shown to be inadequate for the inactivation of *Coxiella burnetii*, which causes Q fever in humans if large numbers are present in raw milk (7). New pasteurization conditions of 62.8°C for 30 min for a batch process or 71.7°C for 15 sec for a continuous process were adopted to inactivate *C. burnetii*, and are still in use today (5).

Based on the commercial pasteurization of cow's milk, originally designed to destroy *M. tuberculosis* and *C. burnetii*, Holder pasteurization (HoP) at 62.5°C for 30 min has been recommended as a suitable form of heat treatment for human milk (8). Interestingly, the recommended temperature has evolved over the years. Thus, in 1999 the United Kingdom Association for Milk Banking (UKAMB) guidelines (9) and in 2000 the Human Milk Bank Association of North America (HMBANA) guidelines (10) recommended that milk be heat-treated at a minimum of 57°C or a maximum of 63°C for 30 min. Despite data showing that this degree of heat is more than adequate to eradicate tuberculosis bacilli from cow's milk and that pasteurization at 62.5°C for 30 min may be excessive for rendering human milk bacteriologically safe (11), HoP is widely recommended in most current guidelines.

This point is especially relevant, as several authors have shown that the processing temperature determines the degree of inactivation of the biological components of milk (12, 13). The effect of pasteurization on the composition of human milk has been recently analyzed in two excellent reviews (14, 15). From a nutritional standpoint, the established heat treatment does not significantly affect the macronutrient composition (protein, carbohydrates and lipids, including polyunsaturated fatty acids) of milk. However, there is considerable evidence for a total eradication of lipase activity, as well as a substantial drop in the concentration of various biological components such as IgA, lactoferrin, lysozyme, cytokines, and growth factors.

The discrepancy about the degree of destruction of these biological factors could be explained by the lack of homogeneity in the experimental studies, which generally use small aliquots of milk compared with the volumes usually processed in HMB. In real-life situations, a higher loss of biological properties is expected due to the longer time needed to reach the desired temperature in the center of the milk container (15). Thus, in the process of securing DHM, there is a reduction in quality due to the destruction of biological components.

The literature reflects the enormous importance of biological factors in milk for the development and maturation of the newborn (16). In an interesting study developed in a preclinical model using premature piglets, Li et al. (17) compared two different treatments of the same DHM: HoP and Ultraviolet (UV)-C irradiation. Analysis revealed a markedly higher reduction of several bioactive proteins after HoP compared with UV-C-treated and untreated milk. The authors reported a better weight gain, intestinal health and resistance against bacterial infections in the group receiving the milk with better preserved bioactive factors. They conclude that the differences in the biochemistry of donor milk due to its treatment have potential physiological effects in preterm neonates. Therefore, one of the goals of HMB should be to process human milk with minimum damage to these components.

## Alternative techniques to HoP

To optimize the microbiological safety of DHM while maintaining its biological and nutritional quality is an important challenge. In this context, the Committee on Nutrition of the European Society for Pediatric Gastroenterology, Hepatology, and Nutrition (ESPGHAN) has recommended that future research focus on the improvement of milk processing in HMB, including the development and evaluation of different pasteurization techniques to optimize microbiological safety and to preserve the biological and nutritional quality of human milk (18). Thus, to avoid the deleterious effect of HoP on the biological components of human milk, attention has been directed toward the development of new technologies. The most evaluated method is high-temperature short-time (HTST) treatment, which involves the heating of milk at 72°C for 15 s. Several reports confirm that this procedure induces a drastic reduction in bacterial count and CMV infectivity. However, although a better preservation of several components like IgA and lactoferrin has been described, bile salt-stimulated lipase (BSSL) activity is almost completely eliminated (19–21). Two alternative prototypes suitable for use in an HMB have been recently described (22, 23).

Pascalization or high pressure processing (HPP) is a method of preserving and sterilizing food in which a product is processed under very high pressure at low temperatures without the use of additives, leading to the inactivation of certain microorganisms and enzymes. Studies with human milk have shown that in comparison with HoP this kind of treatment may increase the retention of IgA, lisozyme and other cytokines (24, 25). A bactericidal effect of HPP has been reported for different microorganisms with varying degrees of resistance (24, 26). However, these studies do not assure the effectiveness of HPP in banked milk with a high bacterial content, especially because only vegetative bacteria have been analyzed and data is lacking about the effects of high pressure on bacterial spores, viruses or fungi in human milk.

UV irradiation treatment is based on the germicidal properties of light in the UV-C spectrum (200–280 nm). UV light penetrates food materials only up to several millimeters, depending on their optical properties, and cannot be effectively absorbed by milk and other turbid foods unless these are presented to the system as a thin layer.

Preliminary reports indicate that UV irradiation can achieve a reduction of 5 log_10_ in bacteria exogenously added to human milk without affecting the lipase activity (27), while the concentrations of lactoferrin, lysozyme and immunoglobulin A (IgA) remain essentially unaltered (28). Also, according to a recent report, UV-C irradiation inactivates the cytomegalovirus in human milk under the right conditions (29).

However, although these techniques are commonly used in the food industry, there are no specific devices designed to manage the low volumes of milk usually processed in a milk bank. Thus, until such appropriately scaled and economically affordable equipment is available for HMB use, these methods are unlikely to be put into practice. Meanwhile, since it has been clearly demonstrated that temperatures below 62.5°C have a less negative effect on human milk properties, it is useful to assess temperature modification in the pasteurization process.

## A need to improve the quality of HMB processing

As early as 1982, Wills et al. (13) proposed that “lower temperatures and reduced holding times, if used precisely, will effectively sterilize human milk. At the same time reduced heat treatment results in the preservation of much of the activity of the antimicrobial and other biologically active proteins present in human milk”.

Thus, a simpler alternative to the development of new technologies would be to optimize the conventional pasteurization technique used in HMB in a way that guarantees the destruction of infectious elements in human milk with minimum damage to its biological components. This option has the advantage that it would not require the replacement of pasteurization equipment usually available in most HMB. Our hypothesis is that the pasteurization conditions currently used are oversized, and that the same level of elimination of bacterial and viral contamination could be achieved using lower temperatures that are less harmful to the biological factors of milk.

Other improvements could also be made in the pasteurization process. The longer the milk remains above the critical temperature, the greater the detrimental effect on its final quality (30). Hence, the quicker the milk can reach the holding temperature, the lower the overall exposure to damaging heat.

## Effect of temperature on bacteria

As mentioned above, most of the studies related to the thermosensitivity of bacteria in milk have been conducted with cow's milk for the dairy industry. Among the few that have focused on human milk from a milk bank perspective, the most detailed analysis was published by Czank et al. (12), who reported that the susceptibility of the microbial strains tested was clearly dependent on the pasteurization temperature. There was a reduction of at least 99.9% of all bacterial species when milk samples spiked with 10^5^ UFC/ml of *E. coli, S. epidermidis, E. cloacae, B. cereus* or *S. aureus* were treated at 57°C for 30 min or at 62.5°C for 20 min. Also, the data of Lloyd Jones et al (31) suggest that the accepted heating time of 30 min is excessive, since bacterial pathogens commonly contaminating human milk may be eliminated after heating for only 5 min at 62.5°C. This conclusion is in accordance with the results of Wills et al. (13), who showed that over 99% of inoculated organisms are destroyed by heating at 56.0°C for 15 min.

Thus, taking into account that the higher the level of milk contamination, the longer it takes to achieve sterility, reducing the temperature of pasteurization would not constitute a hazard in HMB, where highly contaminated milk is discarded. Moreover, in HMB that pool milk, a high level of contamination is diluted.

## Effect of temperature on viruses

In the previous sections, we focused on how the temperature applied in HoP could be considered excessive for the effective removal of bacterial contamination and stability of the main biological components of breast milk. We now turn to viruses and examine if they could also be eliminated at a lower temperature.

In certain maternal viral diseases, there is a substantial risk of maternal-infant transmission by breast milk. This is particularly true for human immunodeficiency virus (HIV)-1, HTLV1/2 and CMV infection. Other viruses are often present in breast milk but transmission is very rare, e.g. other herpes viruses, parvovirus, hepatitis A, B, and C, and rubella (32). It is therefore important that in addition to an accurate anamnesis, including revision of the social, behavioral, and clinical history of the donor, and serological determinations, the donor milk is treated to eliminate possible pathogenic elements.

As mentioned above, pasteurization at 62.5°C may be considered excessive due to its harmful effect on the biological elements in milk. It would therefore be useful to analyse the thermosensitivity of a series of viruses potentially present in donated milk. As it is shown in Table 1, evidence in the literature indicates that most such viruses are destroyed at a temperature between 55 and 60°C. Blumel et al. (33) showed that during pasteurization of human serum, albumin parvovirus B19 was immediately inactivated at temperatures above 57.5°C. Moreover, heating at 55°C for 30 min completely inactivated poliovirus in water and milk (34). Also, incubation of Chikungunya- (CHIKV) or West Nile virus- (WNV) spiked albumin solutions at 58±1°C resulted in a rapid and complete inactivation of both viruses to below the limit of detection within 30 min (35). A very small amount of infectious cell culture-derived Hepatitis C virus (HCV) was still detectable after incubation at 56°C for 30 min, and eliminated completely after 40 min; total viral inactivation was also observed after 10 min at 60°C or 4 min at 65°C (36). Although herpes simplex virus (HSV) has been isolated from the breast milk of HSV-infected women, there is no conclusive evidence to support HSV transmission by breastfeeding (32). In any case, HSV is very sensitive to heat treatment, being inactivated after only 20 min at 50°C (37).

**Table 1:** Viral sensitivity to thermal treatment.

**Virus**	**Pasteurization conditions tested**	**Viral infectivity after treatment**	**Authors**
Parvovirus B19	10′ at 60°C	Completely inactivated	Blümel et al. (33)
Chikungunya virus	30′ at 58 ± 1°C	Completely inactivated	Leydold et al. (35)
Poliovirus	30′ at 55°C	Completely inactivated	Strazynski et al. (34)
West Nile virus	30′ at 58 ± 1°C	Completely inactivated	Leydold et al. (35)
Hepatitis C virus	30′ 56°C	Almost completely inactivated	Song et al. (36)
	40′ at 56°C 10′ at 60°C 4′ at 65°C	Completely inactivated	
Herpes simplex virus	20′ at 50°C	Completely inactivated	Plummer and Lewis (37)
Human Immunodeficiency virus	less than 30′ at 60°C	Completely inactivated	Spire et al. (40) Gregersen et al. (41) Einarsson et al. (42)
Human T lymphotrophic virus	30′ at 56°C	Completely inactivated	Harada et al. (38) Yamato et al. (39)
Zika virus	30′ at 63°C	Completely inactivated	Pfaender et al. (43)
Human papiloma virus	30′ at 62.5°C	Completely inactivated	Donalisio et al. (44)
Ebola virus	30′ at 62.5°C	Completely inactivated	Hamilton Espence et al. (45)
Margburg virus	30′ at 62.5°C	Completely inactivated	Hamilton Espence et al. (45)
Cytomegalovirus	30′ at 62.5°C	Completely inactivated	Hamprecht et al. (47)
	40′ at 50°C	Partially inactivated	Plummer and Lewis (37)
	30′ at 56°C	Partially inactivated	Welsh et al. (46)

HIV and HTLV, potentially the most dangerous viruses, were fully inactivated by treatment at 56°C for 30 min (38–40). Other authors confirmed that at 60°C, HIV in culture supernatants was completely inactivated after only 10 min (41) or after 30 min in stabilized antithrombin III solutions (42).

Another group of viruses are clearly inactivated by conventional HoP conditions but their elimination has not been tested at lower temperatures. Thus, pasteurization of milk spiked with Zika virus (ZIKV) at 63°C for 30 min reduced ZIKV infectivity below the limit of detection, independent of the milk donor or virus strain (43). Also, the infectivity of both high-risk and low-risk human papillomaviruses (HPV) (44) as well as Ebola virus and Marburg virus (45) are completely inactivated after HoP.

Nevertheless, probably the most important virus from the point of view of HMB is CMV. Despite the latency of all herpesviruses, CMV is the only one known to be efficiently transferred to the infant via human milk. There are a few case reports of possible breast milk transmission of HSV and varicella zoster virus (VZV) and strong evidence for the non-transmission of the Epstein-Barr virus (32). Although other members of the herpesvirus group are reported to be destroyed at 50°C, CMV was only partially inactivated at this temperature even after 40 min (37). HoP at 62.5°C for 30 min completely destroyed CMV infectivity in human milk (46, 47), while the treatment of CMV-spiked milk at 56°C for 30 min (46) failed to totally eliminate viral infectivity.

From these data, it could be deduced that a temperature of 62.5°C is required to destroy the CMV, similar to other viruses. Nevertheless, the thermosensitivity of these viruses at temperatures between 56 and 62.5°C has not been reported. Several authors have shown (46, 47) that treatment at 56°C is capable of destroying a significant part of the viral load. Thus, we consider it plausible that the total destruction of the CMV could be achieved at a temperature lower than 62.5°C, and this could be applicable to other viruses not yet tested.

## Future research directions

According to the previous section, we consider that it is feasible to reduce the pasteurization temperature while maintaining the destruction capacity of the bacteria and viruses potentially present in the DHM. Only the thermal sensitivity of the CMV remains to be confirmed, as there is no data in the range between 56 and 62.5°C.

Therefore, we consider it essential to carry out this analysis, accurately determining the CMV sensitivity to thermal treatment. Once established the temperature at which the CMV is inactivated, and confirmed in exact conditions to those used in a HMB, the effect of this temperature on the essential biological components of the DHM (IgA, lipase, lactoferrin, lysozyme…) should be checked. In both studies, it would be interesting to analyse treatment times of less than 30 min, in order to preserve even more the biological properties of the DHM.

## Conclusions

It is clear that donated human milk must undergo treatment to eliminate potentially transmissible pathogenic elements. Unfortunately, a side effect of such processing, including by the widely accepted HoP, is a reduction in the valuable biological properties of the milk. In accordance with the data presented in this review, we propose the assessment of a lower temperature standard for heat treatment of human milk that would be at least the minimum required to eliminate CMV yet less damaging to the biological components. An added advantage of this proposal is its easy implementation in HMB, since the pasteurizers currently in use would not need to be replaced by new equipment.

## Author contributions

AG wrote the initial manuscript. JC edited and finalized the manuscript. All the authors read and approved the final manuscript.

### Conflict of interest statement

The authors declare that the research was conducted in the absence of any commercial or financial relationships that could be construed as a potential conflict of interest.
